# Longitudinal trajectories of internet use, attitudes toward aging, and depression: a latent growth mediation model

**DOI:** 10.3389/fpubh.2026.1853502

**Published:** 2026-08-12

**Authors:** Yunqian Zhou, Jie Qin, Na Cao, Linwei Yu

**Affiliations:** 1 School of Medical Humanities and Management of Wenzhou Medical University, Wenzhou, China; 2 Cixi Biomedical Research Institute, Wenzhou Medical University, Ningbo, China

**Keywords:** aging attitude, China, depression, Internet use, older adults

## Abstract

**Objectives:**

Whether Internet use affects depression in older adults in china via aging attitudes remains unclear. Moreover, few longitudinal studies have been conducted.

**Methods:**

After excluding missing data, the final sample comprised 4,039 older adults (aged ≥60 years) from the 2018, 2020, and 2023 waves of the China Longitudinal Aging Social Survey (CLASS). Using latent growth modeling, we examined the proposed relationship between frequency of Internet use (IU), attitude toward aging (AA) and depression level (DEP) of older adults.

**Results:**

Bootstrapping-based latent growth modeling analyses revealed that the frequency of Internet use and aging attitude of older adults population showed a downward trend, while the level of depression showed an upward trend. The relationships among the three variables are as follows. The initial level of Internet use (IU) indirectly affects the initial level of depression level (DEP) through the initial level of aging attitude (AA) (indirect effect *β* = −0.098, 95% CI [−0.134, −0.068]). The growth rate of Internet use frequency (IU) indirectly affects the growth rate of depression (DEP) through the growth rate of aging attitude (AA) (indirect effect *β* = −0.059, 95% CI [−0.090, −0.040]).

**Conclusion:**

Longitudinal changes in aging attitude have an indirect effect on the relationship between Internet use and depression. It highlights the importance of positive changes in aging attitude and Internet use on depression.

## Introduction

The global aging process is accelerating. According a report from the National Aging Office of the Ministry of Civil Affairs of China, by the end of 2024, China’s older population aged 60 and above had reached 310.31 million, accounting for 22.0% of the total population. However, at the same time, more and more older people are facing health risks such as loneliness, anxiety, and depression (1). Scholars have pointed out that negative emotional states such as loneliness, depression, and social isolation are key aspects of social and psychological loss in later life (2). The detection rate of depressive symptoms among older population in China has reached 20%, and late-life depression has become an important public health issue (3). Depression not only seriously impairs the quality of life and social functioning of older adults (i.e., comorbid physical illness, functional disability, and risk of suicide) (4), but also imposes a heavy burden on families and public healthcare systems. As the population structure ages, the rapid popularization of digital technology has integrated the Internet into daily life, providing new possibilities for older population to maintain social contacts and access resources. According to the China Internet Network Information Center, by 2021, the number of Internet users aged 60 and above in China had exceeded 119 million. This trend has sparked widespread scholarly interest in how Internet use (IU) affects the mental health of this population. Existing studies generally point out that Internet use can help older population to obtain information, maintain social relations and participate in activities, which may reduce the risk of loneliness and depression (5, 6).

Studies suggested that Internet use is negatively associated with depression in older adults (7–9). Longitudinal research has demonstrated that Internet use reduces the probability of a depression state by one third (6). Evidence suggests that among male older adults, Internet use is significantly associated with a decreased risk of depression (9, 10). However, different Internet usage had different effects on depression among older population. Using the Internet for social contact and entertainment decreased the depression scores of the older adults (10, 11). But when using the Internet for learning, work, and commercial activity, the relief of depressive symptoms disappeared (12). According to the theory of social emotional choice, older adults typically prioritize social purposes over informational purposes when using information and communication technology (ICT) (13).

To better understand the complex relationship between Internet use and mental health, it is crucial to examine the psychological mechanisms underlying this link. One such potential mechanism is aging attitude (AA), which refers to the comprehensive system of cognitive evaluation, emotional experience, and behavioral tendencies that individuals hold towards the aging process. It is considered a core psychological construct of positive aging (14). Digital technologies, especially the internet, are also essential facilitators of active aging, offering older adults beneficial experiences and outcomes (15). Internet use can help older adults gain knowledge and skills, boosting self-efficacy and fostering a positive attitudes toward aging (16, 17).

Aging is a process of change that begins at birth and continues until death. It is associated with numerous and inevitable changes in physical, cognitive, and social functioning (18). In the ageing process, individuals’ positive and negative perceptions of aging are crucial for coping with the accompanying physical, psychological, and social changes, and may even influence their longevity (19–21). Research has showed n that individuals who hold a more negative attitude towards aging experience more severe symptoms of depression, cognitive decline, and even premature death (22, 23). Internalized ageism can contribute to feelings of inadequacy, depression, or anxiety, as the individual may feel they do not meet societal expectations of youth or vitality (2). More positive attitudes toward aging were associated with higher levels of life satisfaction, better self-reported physical and mental health, and lower levels of anxiety and depression (24, 25). Older adults with a positive aging attitude are more likely to engage in preventive health behaviors (26).

The existing literature either examines the relationship between Internet use and depression, or focuses on aging attitude and its influence mechanism, or treats aging attitude as an independent variable affecting depression. Most of them are cross-sectional studies. In order to deeply analyze the internal mechanism and complex longitudinal dynamics between Internet use frequency and depression level, this study adopted latent growth modeling (LGM). This study explored the trajectory of changes in aging attitude to test the longitudinal mediation of its initial level and growth rate on the association between Internet use and depression. The core research questions of this study are as follows:

*RQ1*: The initial level and growth rate of Internet use directly affect the initial level and growth rate of depression among older adults.

*RQ2*: The initial level and growth rate of Internet use influence the initial level and growth rate of depression by affecting the initial level and growth rate of aging attitude among older adults.

## Methods

### Data and samples

This study used data from three waves of the China Longitudinal Aging Social Survey (CLASS), covering a 5-year period from 2018 to 2023. The CLASS dataset includes information on multiple domains, including personal basic information, health and related services, socio-economic status, the older adults care planning and social support, psychological feelings, family and children, daily activities and fitness. Preliminary data examination suggested that including the earlier waves would lead to substantial sample attrition due to loss to follow-up. Accordingly, to maximize the available sample size and maintain sufficient statistical power for the latent growth model, we restricted the analysis to the 2018–2023 waves. CLASS adopts stratified and multi-stage probability sampling method, selects county-level regions (including counties, county-level cities and districts) as primary sampling units, and village/neighborhood committees as secondary sampling units, focusing on older persons aged 60 and above in Chinese Mainland, covering 28 provinces, and more details can be accessed http://class.ruc.edu.cn/.

Based on the aforementioned research question, this article will limit the analysis to respondents aged 60 and above at the time of the initial survey. In order to effectively test the trend of Internet use frequency and depression level of the surveyed older adults over time and whether older adults attitude towards aging plays a mediating role, this paper further limits the analysis objects to the samples that all participated in the survey in 2018, 2020 and 2023. After screening, after excluding participants with incomplete data on Internet use frequency, aging attitude and depression, 4,039 participants remained in the final analysis.

### Dependent variable

Depression was assessed using the 9-item CES-D scale revised by Silverstein (27). Two items reflect negative emotions (loneliness and depression), and feelings of marginalization (being idle and feeling useless), and one item reflects somatic symptoms (difficulty sleeping and loss of appetite). In addition, three items represent positive emotions: pleasure, enjoyment of life, and happiness. Each item is assigned a value of 0 (never), 1 (sometimes) or 2 (often). These ratings reflect how often participants experienced each item per week. Reversing the coding of the forward items, we summed the scores of the nine items, yielding depressive symptom scores ranging from 0 to 18. Higher scores suggested more severe depression.

### Independent variable

The questionnaire includes a question asking respondents, ‘Do you use the internet (including various electronic devices such as mobile phones)?’. Internet use was assessed on a 5-point scale (1 = ‘daily use’ to 5 = ‘never use’). To facilitate interpretation, we reverse-coded the scale so that higher scores represent more frequent Internet use. All analyses reported in this manuscript are based on the reverse-coded scores.

### Mediating variables

Aging attitude was measured using the AAQ (Aging Attitude Questionnaire) (28). This scale consists of 8 items and is scored using a 5-point Likert scale, ranging from “completely disagree” to “completely agree.” Four of the items (e.g., “If given the opportunity, I would be happy to participate in some work of the village/community committee”), assess positive attitudes towards aging, with higher scores indicating more positive attitudes. The other four items (e.g., “Social changes are so fast that I find it difficult to adapt to these changes”) assess negative attitudes, and their scores also reflect more positive attitudes after reverse scoring. Total scores range from 8 to 40 points, with higher scores indicating a more positive attitude towards aging.

### Covariates

Previous studies have showed that, at the individual level, the factors influencing the frequency of Internet use and depression of older adults mainly include gender, age, education, marital status, household registration, physical health, etc. This article selects these factors as control variables to be included in the model, in order to eliminate possible confounding factors as much as possible. Physical health status was measured using the Activities of Daily Living (ADL) scale. Respondents were asked whether they had difficulties in nine daily activities, including going up and down stairs, whether they had fallen, whether they could walk outside, whether they could use public transportation independently, and whether they could go out shopping.

### Statistical analysis

Use Stata 17.0 software for data organization, generation of descriptive statistics, and correlation analysis. Before conducting the analysis, we established a structural equation model between variables using Mplus 8.0 through three steps. Firstly, a linear growth model (LGM) was used to examine the development trajectories of each variable. After extracting the intercept and slope, the intercept represented the initial level of the variable (intercept factor loading set to 0, 2, 5), and the slope represents the growth rate of the variable (slope factor loading set to 0, 2, 5). Secondly, a conditional LGM was fitted using three measurements of Internet use frequency and depression level to test the direct effect of Internet use frequency on the intercept and slope of depression variables. Finally, a structural equation model was established among the frequency of Internet use, aging attitude and depression to estimate direct paths of the intercept and slope of each variable. Model fit was evaluated using several common indicators: the ratio of the chi-square statistic to its degrees of freedom (*χ*^2^/df), the root mean square error of approximation (RMSEA), comparative fit index (CFI), Tucker Lewis index (TLI), etc. A good model fit was suggested by *χ*^2^/df < 5, RMSEA < 0.08, and both CFI and TLI > 0.90. In addition, based on the model establishment, the significance of the mediating effect was verified using a bootstrap method (1,000 resamples). If the confidence interval (CI) does not include 0, then the effect is statistically significant. When controlling for the influence of demographic variables, baseline variables such as age, gender, education level, marital status, cohabitation status, household registration type, and daily living activity ability are used as time-invariant covariates to be included in the model, in order to adjust for potential confounding effects of these variables on the initial level and development rate.

## Results

### Descriptive analysis

Table 1 presents the baseline characteristics of the sample, including the means and standard deviations of the key variables over time. The average age of the sample is 72.24 years, with males accounting for 50.53% and participants with non-agricultural hukou accounting for 46.67%. Other demographic characteristics include: 82.4% are married, and 61.15% have a primary school education or below. The baseline mean score for the assessment of daily living activity ability of the sample is 9.487. The score of Internet use frequency showed an increasing trend year by year. On the contrary, the average score of aging attitude showed a downward trend. The level of depression increased from 2018 to 2020, but showed a downward trend again by 2023.

**Table 1:** Socio-demographic characteristics of participants.

Variables	Time 1		Time 2		Time 3	
	*N*	M ± SD (%)	*N*	M ± SD (%)	*N*	M ± SD (%)
Age	4,039	72.24 ± 4.65				
Gender (male)	2041	50.53				
female	1998	49.47				
Married	3,328	82.4				
Non-married	711	17.6				
Education (illiterate/semi-literate)	1,017	25.18				
Primary	1,453	35.97				
Junior high	1,094	27.09				
High school/secondary specialized or above	475	11.76				
Hukou (rural)	2,154	53.33				
Urban	1885	46.67				
Coresidents(1)	358	8.86				
2	2,597	8.86				
3	284	7.03				
4	326	8.07				
5	380	9.41				
6	74	1.83				
7	13	0.32				
8	5	0.12				
9	1	0.02				
10	1	0.02				
ADL	4,039	9.487 ± 1.557				
Internet use	4,039	0.884 ± 1.587	4,039	1.096 ± 1.707	4,039	1.483 ± 1.845
Aging attitude	4,039	16.784 ± 4.196	4,039	16.502 ± 4.095	4,039	16.354 ± 4.013
Depression	4,039	6.559 ± 3.096	4,039	6.695 ± 3.205	4,039	6.588 ± 3.273

ADL, Activity of Daily Living. Data presented are means (standard deviations) or number (%), as appropriate for variables.

### Trajectories of IU, AA and DEP

We used an unconditional linear growth model (LGM) to analyze the development trajectories of the research variables, and fit IU, AA, and DEP separately. Table 2 lists the fit indices and mean intercepts and slopes of three models. The study found that the models of IU, AA, and DEP fit the data well, and their mean slopes are positive, indicating that the development of these three variables showed a linear growth trend.

**Table 2:** LGM fitting indicators, intercepts, and slopes of key variables.

Variables	*χ* ^2^	df	RMSEA	CFI	TLI	MEAN	
						Intercept	Slope
IU	2.809	1	0.021	1.000	0.999	0.877	0.161
AA	7.380	1	0.040	0.999	0.996	16.746	1.191
DEP	13.160	1	0.055	0.997	0.992	6.599	0.422

***p < 0.001. IU, internet use frequency; AA, aging attitude; DEP, depression.

### Direct effect of IU on the initial level and growth rate of DEP

**Figure 1:**
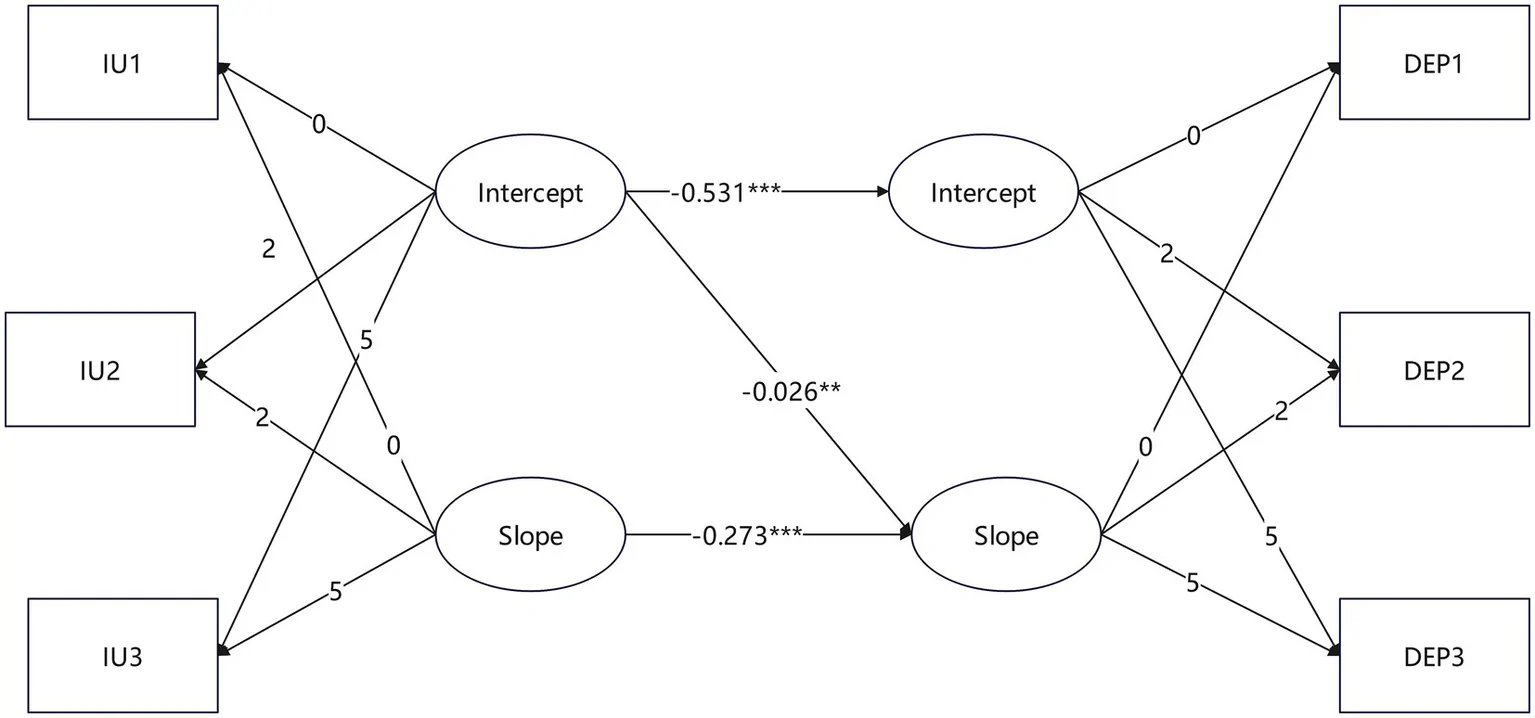
Conditional LGM with *IU* as independent variable. *p < 0.05, ***p < 0.001. IU, internet use frequency; AA, aging attitude; DEP, depression; T1, T2, and T3, respectively, signifies 2018, 2020, and 2023.

We constructed a conditional LGM with IU as the independent variable and DEP as the dependent variable, aimed to investigate whether IU affects the initial level and growth rate of DEP. Figure 1 simplified path diagram. This model adopted path analysis, using standardized path coefficients of key variables as analysis indicators. The fitting effect of the conditional model was good (*χ*/df = 215.116/21, CFI = 0.984, TLI = 0.957, RMSEA = 0.048). IU intercept was a significant negative associated with DEP intercept (*β* = −0.531, *p* < 0.001). The slope also showed a significant negative impact (*β* = −0.026, *p* < 0.05). This suggested that a higher initial level of IU was associated with a lower initial level of DEP, and the slower the growth rate of DEP. The slope of IU was a significant negative associated with the slope of DEP (*β* = −0.273, *p* < 0.001). This suggests that more frequent Internet use among older adults may be associated with a slower increase in depression levels.

### Longitudinal mediation of AA between IU and DEP

**Figure 2:**
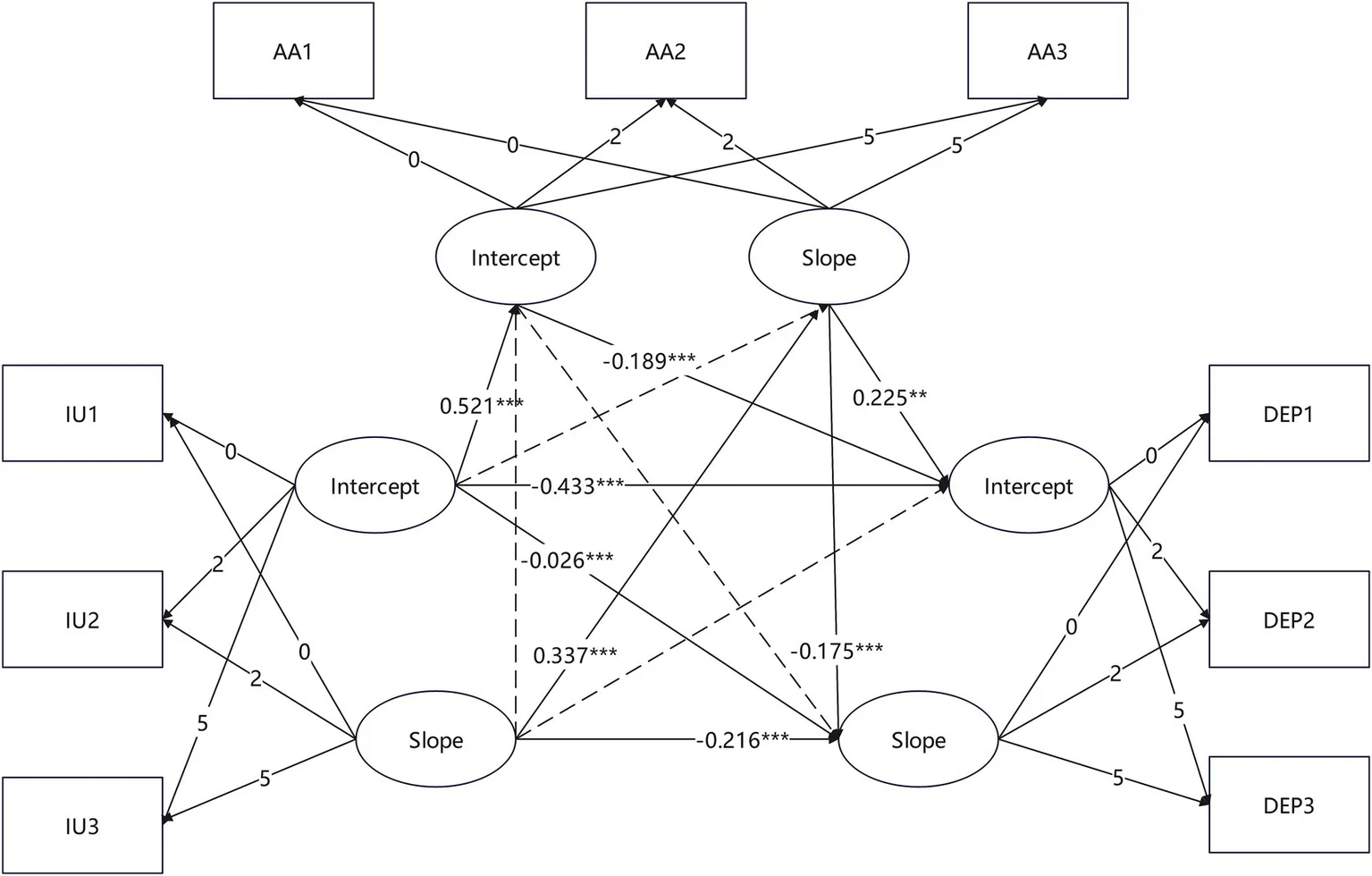
Longitudinal mediation model with *AA* as mediator. *p < 0.05, ***p < 0.001. IU, internet use frequency; AA, aging attitude; DEP, depression; T1, T2, and T3, respectively, signifies 2018, 2020, and 2023.

We used latent growth models (LGM) for IU, AA, and DEP to test the longitudinal mediating effect of AA. By establishing a structural equation model and testing its fitting indicators to verify the mediating effect of AA, the results showed that the model fitted the data well (*χ*^2^/df = 401.429/39, CFI = 0.975, TLI = 0.936, RMSEA = 0.048), and Figure 2 showed the final model.

### Direct effects between the variables in the longitudinal mediation model

The table showed the direct path between IU, AA, and DEP. After controlling for covariates such as gender, age, household registration, education level, marital status, cohabiting status, and daily living ability, the IU intercept was positively associated with the AA intercept (*β* = 0.521, *p* < 0.001). This result suggested that a higher initial level of IU may have a positive effect on the initial level of AA. In addition, the slope of IU was positively associated with the slope of AA (*β* = 0.337, *p* < 0.01). The growth rate of IU was positively associated with the growth rate of AA. Further analysis revealed that the intercept of IU was significantly and negatively associated with the intercept of DEP (*β* = −0.433, *p* < 0.001). The IU intercept also significantly negatively predicted the slope of DEP (*β* = −0.026, *p* < 0.01). This suggests that a higher initial level of IU may be associated with a lower initial level of depression and a slower growth rate of depression over time. The AA intercept was also significantly and negatively associated with the DEP intercept (*β* = −0.189, *p* < 0.001), indicating that a higher initial level of AA is associated with a lower initial level of depression in older people. Ultimately, the IU slope (*β* = −0.216, *p* < 0.01) and AA slope (*β* = −0.175, *p* < 0.001) were both significantly and negatively associated with the DEP slope, suggesting that the growth rates of IU and AA were associated with a slower growth rate of DEP.

### Test of the longitudinal mediation effect of AA

We used a bootstrap method (1,000 resamples) to verify the mediating effects of intercept and slope of AA, in order to explore the longitudinal impact of AA on the association between IU and DEP. The model includes the following indirect paths: (1) IU intercept → AA intercept → DEP intercept, (2) IU slope → AA slope → DEP slope. The results of the bootstrap method showed that these two indirect paths are significant (see Table 3), indicating that the initial level and growth rate of AA may serve as longitudinal mediators between the frequency of Internet use and the level of depression of older persons. Among them, AA intercept partially mediates the relationship between IU intercept and DEP intercept (*β* = −0.098, 95% CI [−0.134, −0.068]), explaining 18.3% of the total effect, indicating that the frequency of Internet use at a higher initial level was associated with a higher initial level of aging attitude, which in turn may be associated with a lower initial level of depression in older adults. In addition, the slope of IU partially mediates the correlation with the slope of DEP through the slope of AA (*β* = −0.059, 95% CI [−0.090, −0.040]), and its explanatory power accounts for 21.4% of the total effect, indicating that a faster growth rate of Internet use may be positively associated with the growth rate of aging attitude, which in turn may be associated with a slower growth rate of depression in older adults.

**Table 3:** Indirect paths in the longitudinal mediation model.

Indirect paths	Indirect effect	*p*	95% Bootstrap		Proportion of mediating effect
IUintercept → AAintercept → DEPintercept	−0.098	0.000	−0.134	−0.068	18.5%
IUslope → AAslope → DEPslope	−0.059	0.000	−0.090	−0.040	21.4%

IU, internet use frequency; AA, aging attitude; DEP, depression.

## Discussion

Three groups of unconditional LGMs revealed that IU, AA, and DEP all showed significant linear increasing trends over time. The average slope of IU is positive and significant, indicating that Internet use among older adults has increased during the study period (13). This finding is consistent with the statistics of the China Internet Information Center, and showed that digital technology continues to penetrate into the daily life of older population. The positive slope of DEP suggested a linear increase over time, which means that the risk of depression gradually increased with age (29–31).

Also our research established a conditional LGM, and the model results showed that IU has a significant direct association on DEP, both in the initial state and development trajectory. First, the intercept of IU was negatively correlated with the intercept of DEP, indicating that older people who used the Internet more frequently at baseline reported fewer depressive symptoms. Numerous cross-sectional studies have confirmed this (5, 7, 32). A systematic review included 18 quantitative studies, of which 7 supported the significant role of Internet use in reducing depression symptoms (33). Secondly, we found that the intercept of IU negatively predicted the slope of DEP. As time goes by, older persons who used the Internet more frequently at the beginning increased their depressive symptoms more slowly. Early Internet use seems to have a lasting protective effect, which may buffer the progress of age-related depression. Furthermore, the slope of IU is negatively correlated with the slope of DEP. This suggests that over time, the speed of older population’s increasing Internet use was associated with the speed of their increasing depressive symptoms. Those with faster growth in Internet use tended to experience a slower increase in depression. This dynamic longitudinal relationship better demonstrates protective effects than individual cross-sectional associations, as it captures the co-evolution of these behaviors and mental health outcomes over time.

We further constructed a multivariate latent growth model to examine whether attitudes towards aging (AA) play a longitudinal mediating role. The results demonstrated that AA operated through two different but interrelated pathways: one involves the initial level of variables (intercept), and the other involves their rate of change over time (slope).

The first indirect pathway suggested that the intercept of IU significantly predicts AA’s intercept, which in turn significantly predicts DEP’s intercept. This finding showed that older people who use the Internet more frequently at baseline tend to have a more positive attitude towards their own aging, which is related to the lower level of initial depression symptoms. The Internet enabled older population to access information that challenges age stereotypes (15), master digital skills, which enhances self-efficacy (16, 17). and the value identity brought by online social networking (14). Subsequently, the negative correlation between AA and DEP at the initial level is consistent with a large body of literature, which suggests that how individuals perceive their aging may profoundly affect their mental health. A positive attitude towards aging is associated with better coping strategies, more preventive health behaviors, and higher life satisfaction, all of which may prevent depression (24–26). On the contrary, internalized negative age stereotypes may lead to feelings of inadequacy and helplessness, ultimately resulting in symptoms of depression (19).

The second indirect pathway suggested that the slope of IU significantly predicts the slope of AA, which in turn significantly predicts the slope of DEP. The mediating effect of AA slope on the relationship between IU slope and DEP slope is significant. This showed that older adults with faster development of Internet use also have faster improvement in their attitude towards aging, which showed down the development of depression. As time goes by, older people become more and more frequent and skilled Internet users, and they may gradually expand their online activities - from simple communication to more diversified activities, such as information seeking, entertainment, and even lifelong learning (16, 34, 35). Every new mastery of skills further enhances self-efficacy, challenges assumptions of age discrimination, and forms a reinforcing cycle of positive self-perception (16). On the contrary, those who do not increase Internet use may miss these cumulative benefits. The attitude towards aging is not a fixed characteristic, but rather evolves with life experiences and interventions (14). Our research findings suggested that the increasing integration of digital technology into daily life is a transformative experience. Over time, older adults have a more positive view of aging and may become more resilient to stress and loss, which often lead to depression. They may be more likely to engage in behaviors that promote health, maintain social connections, and adopt proactive coping strategies.

In general, these two intermediary approaches provide a comprehensive picture of how attitudes towards aging play a role as a mechanism linking Internet use and mental health. The mediation mechanism reveals the differences between people established at the beginning. People who are already Internet users have formed a more positive aging attitude and better mental health than non-Internet users. The mediating mechanism of slope suggested that people’s internal development process unfolds over time. As individuals strengthen Internet use, their aging attitudes are improved, and their mental health trajectory is also benefited accordingly. Aging perceptions may be a modifiable factor to target for future interventions (20). Clinicians might need to consider attitudes toward aging along with social support as they work with older adults with comorbid physical illnesses and depression (36).

This study has several limitations. First, although the longitudinal design is adopted, observational study cannot fully establish the causal relationship, and the association between Internet use and depression may be affected by other variables. Secondly, the core variables are measured using self-reported scales, which may lead to recall bias. Future research can combine objective data to verify this. Thirdly, this study focuses on the frequency of Internet use, and does not distinguish between specific purposes of use and content. However, existing studies showed that different use purposes may have differential effects on mental health, and subsequent studies need to further refine this dimension. Finally, we were unable to fully examine the specific effects of the digital divide on particularly vulnerable subgroups of older adults, such as those living in rural areas and those who are unmarried. These groups may face multiple barriers—including limited internet access and lack of social support—that place them at a greater disadvantage in the relationship between internet use and mental health. Future research should collect more targeted samples and design tailored interventions for these subgroups.

Interventions should target disadvantaged subgroups who face multiple barriers to digital inclusion and its mental health benefits. Additionally, the research results of mediation mechanism showed that promoting Internet use among older adults who do not use the Internet at present may benefit their aging attitude and mental health. However, the slope mediation results suggested that simply allowing older adults to access the internet is not enough. Over time, supporting them to increase and diversify their participation may be beneficial in improving older adults’ attitudes towards aging and depression. Therefore, intervention measures should not only focus on initial access and training, but also on providing sustained support and introducing new online opportunities to sustain the engagement and skill development in the digital domain. Mandatory age-friendly design standards for public-facing apps and websites should be enforced, including larger fonts, voice interaction, and one-click simplified modes, while low-cost smart devices such as one-button emergency call systems should be subsidized for disadvantaged older adults. Community-based digital learning hubs should be established in villages and urban communities, offering free training through peer-support models (e.g., “younger seniors helping older seniors”) and volunteer networks. Dedicated legislation should protect older adults’ digital rights by requiring accessibility certification for digital products, and financial incentives like subsidies or tax breaks should be provided to encourage age-friendly design. Public awareness campaigns should challenge ageist stereotypes about technological adoption, promote digital safety education to reduce scam-related fears, and encourage intergenerational “digital back-feeding” as a social norm.

## Data Availability

The data analyzed in this study were obtained from the China Longitudinal Aging Social Survey (CLASS), conducted by Renmin University of China. Data are available upon reasonable request to the CLASS project team via class.ruc.edu.cn.
